# Correlates of depressive symptoms in late middle-aged Taiwanese women: findings from the 2009 Taiwan National Health Interview Survey

**DOI:** 10.1186/s12905-017-0461-4

**Published:** 2017-11-09

**Authors:** Kun-Wei Tsai, Shih-Chun Lin, Malcolm Koo

**Affiliations:** 1 Division of Geriatrics, Dalin Tzu Chi Hospital, Buddhist Tzu Chi Medical Foundation, Dalin, Chiayi Taiwan; 2 Department of Medical Research, Dalin Tzu Chi Hospital, Buddhist Tzu Chi Medical Foundation, Dalin, Chiayi Taiwan; 3 Dalla Lana School of Public Health, University of Toronto, Toronto, ON Canada

**Keywords:** Menopausal symptoms, Climacteric symptoms, Depressive disorder, Middle-aged, Health surveys

## Abstract

**Background:**

Previous studies have shown that depressive symptoms in middle-aged women were associated with a number of factors such as climacteric symptoms. Nevertheless, studies based on population-based data with a wide range of potential correlates are still scarce. Therefore, the aim of this study was to investigate the correlates of depressive symptoms in late middle-aged Taiwanese women using data from a nationally-representative, population-based survey.

**Methods:**

Women aged 50.0–65.0 years were identified from the dataset of the 2009 Taiwan National Health Interview Survey. The outcome measure was depressive symptoms in the past week, evaluated using the Center for Epidemiologic Studies Short Depression Scale (CES-D 10) with a cut-off score of 10 or greater. Univariate and multiple logistic regression analyses were used to evaluate the correlates of depressive symptoms.

**Results:**

The mean age of the 533 respondents was 56.7 years. Depressive symptoms were present in 53 respondents (9.9%). Multiple logistic regression analysis revealed that an education level of elementary school or below (adjusted odds ratio [AOR] = 3.19, *P* = 0.003), nulliparity (AOR = 8.10, *P* = 0.001), living alone (AOR = 5.47, *P* = 0.003), never having worked (AOR = 4.14, *P* = 0.008), lack of regular exercise (AOR = 3.01, *P* = 0.003), a perceived health status of fair or bad (AOR = 4.34, *P* < 0.001), and somatic climacteric symptoms (AOR = 2.32, *P* = 0.012) were independent and significant factors of depressive symptoms in late middle-aged Taiwanese women.

**Conclusions:**

Findings from this secondary analysis of a population-based survey suggested independent associations of somatic climacteric symptoms, and a number of socio-demographic and health-related factors with depressive symptoms in late middle-aged Taiwanese women.

## Background

Depression is an important public health problem [1] because of its association with suicidal ideation [2], poor self-rated health [3], increased use of health services [4], and mortality [5]. Depression can also increase the risk of mortality in patients with cancer [6] and established coronary disease [7]. Epidemiological studies reveal that increased age, being of the female sex, and being separated or divorced in terms of marital status are associated with higher rates of depression [8]. Moreover, depression is prevalent among patients with serious chronic medical conditions, such as cancer, cardiovascular disease, chronic obstructive pulmonary disease, and type 2 diabetes [9–12]. The likelihood of being in a depressed mood during the transition to menopause ranged from 1.3–3 times greater than that during premenopause [13]. The transition to menopause appears to be a “window of vulnerability” for the development of depressive symptoms in some women [14]. Therefore, increased risk of depression is an important health issue for middle-aged women.

While a number of studies have investigated the relationship between climacteric symptoms and depressive symptoms [15–17], relatively few have focused on Chinese women. A cross-sectional, community-based survey of 393 middle-aged women in Hong Kong found that a marital status of not married or not cohabiting with a partner, a lower educational level, having multiple chronic diseases, and having experienced certain stressful life events were significant factors associated with depressive symptoms [18]. Another cross-sectional study of 672 Taiwanese aboriginal women aged between 40 and 60 years showed that a higher score for physiological postmenopausal symptoms was significantly associated with depression [19]. Moreover, a cross-sectional study based on a convenience sample of 566 women from a medical center and residential community in southern Taiwan reported that menopausal symptoms, attitudes towards menopause and aging, family income, and chronic disease status were significantly associated with depressive symptoms [20]. The only population-based study on depression in midlife Taiwanese women was a secondary data analysis of the 2002 Health Promotion Knowledge, Attitude, and Performance Survey. The study reported that perimenopause was significantly associated with depression in midlife women, after adjusting for age, marital status, education, income, smoking, hormone therapy and menopausal symptoms [21]. Therefore, the aim of this study was to investigate the correlates of depressive symptoms among late middle-aged women using data from a nationwide, population-based survey of Taiwan.

## Methods

### Study design and data source

The data for this cross-sectional study were obtained from the Taiwan National Health Interview Survey (NHIS) conducted in 2009. The NHIS is a nationally-representative survey of the total population of Taiwan conducted by the Health Promotion Administration of the Ministry of Health and Welfare once every 4 years.

Individuals in the NHIS were sampled using a multistage, stratified, systematic sampling design based on geographic location and level of urbanization. All selected individuals were interviewed by trained interviewers face-to-face using computer-assisted personal interviewing (CAPI). Further details regarding questionnaire content and sampling design can be obtained from the NHIS website (http://nhis.nhri.org.tw/). A total of 30,528 (1.33%) eligible individuals were sampled from the Taiwanese population of 22,942,706 as of December 31, 2008. A total of 25,632 individuals completed the survey (84.0% response). Of them, 2441 were females aged 50.0 to 65.0 years. A random sample of 638 respondents was further selected using the CAPI software to respond to a set of optional questions including those on depressive symptoms, which was the main outcome of interest in this study. Eighteen of these respondents had missing data for height or weight and 42 respondents had missing data on menopausal symptoms. As a result, a total of 578 women had complete data for all variables. After weights were applied to the raw data, a final sample of 533 women was available for analysis.

The study protocol was reviewed and approved by the institutional review board of the Dalin Tzu Chi Hospital, Buddhist Tzu Chi Medical Foundation, Taiwan (No. B10204005). Since the datafile contained only de-identified secondary data, the institutional review board waived the requirement for informed consent.

### Measure of depressive symptoms outcome

The outcome variable of this study was depressive symptoms in the week preceding the interview, measured by a 10-item Chinese version of the Center for Epidemiologic Studies Short Depression Scale (CES-D 10). The original 20-item version of the CES-D was developed in English in 1977 [22]. The 10-item brief version has been shown to adequately represent the full CES-D [23]. The psychometric properties of the Chinese version have been evaluated in elderly Chinese. Satisfactory content reliability and temporal reliability were attained with accuracy in classifying individuals with depressive symptoms and they were comparable to the original CES-D. A good internal consistency of Cronbach’s α = 0.78 and a moderate test-retest reliability at *r* = 0.44 over 3 years were observed [24]. The CES-D 10 comprises 10 questions with a four-point Likert scale graded as follows: rarely or none of the time = 0 (less than a day per week); some of the time = 1 (1 or 2 days per week); occasionally = 2 (3 or 4 days per week); and almost all the time = 3 (5 to 7 days per week), with a total score of 30. Two items (numbers 5 and 8) required reverse scoring. Respondents were classified as having depressive symptoms if they had a total score of 10 or above [25]. A confirmatory factor analysis of the Chinese CES-D 10 was conducted on a sample of 742 community-dwelling Chinese elderly aged 60 years and older. A two-factor model that divided items into “depressive affect/somatic retardation” and “positive affect” constructs were found to best fit the data. The reliability coefficients also revealed that most indicators were strongly related to their purported latent factors (range from 0.46–0.89) [26].

### Measures of independent variables

A total of 20 independent variables were evaluated in this study, including age, body mass index, educational level, marital status, nulliparity, living arrangements, religious belief, monthly personal income, work status, smoking habit, alcohol consumption, regular exercise, hypertension, hyperlipidemia, diabetes, perceived health status, use of emergency services in the past year, hospitalization in the past year, experience with any somatic climacteric symptoms, and experience with any psychological climacteric symptoms.

Age was categorized into 50.0–54.9, 55.0–59.9, and 60.0–65.0 years. Body mass index was categorized as normal, underweight, preobese, and obese, using the cut-off criteria set by the Ministry of Health and Welfare in Taiwan. Educational level was dichotomized into secondary school or above and elementary school or below. Marital status was dichotomized as married versus other, which included single, divorced, separated, and widowed. Work status was categorized into currently working, having worked in the past, and never having worked, with work defined as employment with paid compensation. Smoking habit was defined as either non-smokers or daily and occasional smokers. Alcohol consumption was defined as the consumption of alcoholic beverages in the past month. Regular exercise was defined as having engaged in any physical activity in the past 2 weeks. The presence of hypertension, diabetes, and hyperlipidemia were determined by two sequential questions: “Do you have this disease?” and “Were you told by a doctor or a nurse that you have this disease?” Only if the answers to both questions were affirmative, then the patient was classified as having the disease. The somatic climacteric symptoms and psychological climacteric symptoms were both dichotomous variables. A respondent was considered as having somatic climacteric symptoms if she had ever experienced any of the following 14 symptoms: hot flashes, night sweats, palpitations, chest tightness, dizziness, headache, vaginal dryness, loss of libido, dyspareunia, insomnia, muscle or joint ache, fatigue, dry skin, and other somatic changes. Moreover, a respondent was considered as having psychological climacteric symptoms if she had ever experienced any of the following 6 symptoms: anxiousness, dysphoria, panic attacks, feeling depressed, forgetfulness, and other psychological changes.

### Statistical analysis

Univariate logistic regression analyses were used to determine the odds ratio (OR) and 95% confidence interval (CI) of the risk of depressive symptoms associated with the explanatory variables. In addition, multiple logistic regression analysis was used to assess the independent association between depressive symptoms and the 20 explanatory variables using a backward elimination procedure, based on the likelihood ratio test statistic. The data were weighted by age and sex to achieve a nationally representative sample. A two-tailed *P* value of <0.05 was considered statistically significant. All statistical analyses were conducted using the IBM SPSS Statistics software package, version 24.0 (IBM Corp, Armonk, NY, USA).

## Results

The mean age of the 533 women in this study was 56.7 years (standard deviation = 3.8 years). Table 1 shows the distribution of the 20 independent variables in the 53 (9.9%) and 480 (90.1%) women with and without depressive symptoms, respectively. Results of the univariate logistic regression analyses indicated that an educational level of elementary school or below (OR = 4.18, *P* < 0.001), a marital status of other than being married (OR = 2.12, *P* = 0.015), nulliparity (OR = 6.19, *P* < 0.001), living alone (OR = 2.70, *P* = 0.030), never having worked (OR = 5.33, *P* = 0.001), lack of regular exercise (OR = 4.32, *P* < 0.001), hypertension (OR = 1.86, *P* = 0.041), diabetes (OR = 2.57, *P* = 0.014), a fair or bad perceived health status (OR = 5.18, *P* < 0.001), hospitalization in the past year (OR = 2.35, *P* = 0.037), and somatic climacteric symptoms (OR = 1.89, *P* = 0.029) were significantly associated with depressive symptoms.

**Table 1:** Univariate logistic regression analysis of depressive symptoms in Taiwanese women aged 50 to 65 years (*N* = 533)

Variable	Frequency (%)			Odds ratio (95% CI)	*P* value
	Total	Depressive symptoms	No depressive symptoms		
	533 (100)	53 (9.9)	480 (90.1)		
Age category (years)					
50.0–54.9	200 (37.5)	14 (26.4)	186 (38.8)	1.00	
55.0–59.9	206 (38.6)	24 (45.3)	182 (37.9)	1.83 (0.91–3.65)	0.089
60.0–65.0	127 (23.8)	15 (28.3)	112 (23.3)	1.85 (0.86–4.00)	0.117
Body mass index					
Normal	249 (46.7)	25 (46.3)	224 (46.8)	1.00	
Underweight	19 (3.6)	0 (0)	19 (4.0)	0.10 (0.001–7.14)	0.293
Preobese	163 (30.6)	20 (37.0)	143 (29.9)	1.25 (0.67–2.35)	0.480
Obese	102 (19.1)	9 (16.7)	93 (19.4)	0.84 (0.37–1.90)	0.678
Educational level					
Secondary school or above	288 (54.0)	13 (24.5)	275 (57.3)	1.00	
Elementary school or below	245 (46.0)	40 (75.5)	205 (42.7)	4.18 (2.17–8.05)	<0.001
Marital status					
Married	417 (78.2)	34 (64.2)	383 (79.8)	1.00	
Other	116 (21.8)	19 (35.8)	97 (20.2)	2.12 (1.16–3.89)	0.015
Nulliparity					
No	515 (96.4)	46 (86.8)	469 (97.5)	1.00	
Yes	19 (3.60)	7 (13.2)	12 (2.5)	6.19 (2.31–16.58)	<0.001
Living arrangement					
Not living alone	501 (94.0)	46 (86.8)	455 (94.8)	1.00	
Living alone	32 (6.0)	7 (13.2)	25 (5.2)	2.70 (1.10–6.61)	0.030
Religious belief					
No	63 (11.8)	6 (11.3)	57 (11.9)	1.00	
Yes	470 (88.2)	47 (88.7)	423 (88.1)	1.04 (0.43–2.54)	0.925
Monthly personal income (NT$^a^)					
≥ 20,000	181 (35.4)	13 (24.5)	168 (36.6)	1.00	
< 20,000	331 (64.6)	40 (75.5)	291 (63.4)	1.73 (0.90–3.31)	0.100
Work status					
Working now	240 (44.9)	18 (34.0)	222 (46.2)	1.00	
Worked in the past	266 (49.8)	27 (50.9)	239 (49.7)	1.41 (0.75–2.63)	0.286
Never worked	28 (5.2)	8 (15.1)	20 (4.2)	5.33 (2.08–13.70)	0.001
Smoking habit					
No	513 (96.2)	49 (92.5)	464 (96.7)	1.00	
Daily or occasionally	20 (3.8)	4 (7.5)	16 (3.3)	2.45 (0.81–7.42)	0.112
Alcohol consumption in the past month					
No	470 (88.2)	46 (86.8)	424 (88.3)	1.00	
Yes	63 (11.8)	7 (13.2)	56 (11.7)	1.22 (0.54–2.79)	0.631
Regular exercise					
Yes	309 (57.9)	14 (26.4)	295 (61.3)	1.00	
No	225 (42.1)	39 (73.6)	186 (38.7)	4.32 (2.29–8.14)	<0.001
Hypertension					
No	394 (73.8)	33 (62.3)	361 (75.1)	1.00	
Yes	140 (26.2)	20 (37.7)	120 (24.9)	1.86 (1.03–3.35)	0.041
Hyperlipidemia					
No	405 (76.0)	38 (71.7)	367 (76.5)	1.00	
Yes	128 (24.0)	15 (28.3)	113 (23.5)	1.33 (0.71–2.49)	0.381
Diabetes					
No	482 (90.4)	43 (81.1)	439 (91.5)	1.00	
Yes	51 (9.6)	10 (18.9)	41 (8.5)	2.57 (1.21–5.45)	0.014
Perceived health status					
Extremely good, very good, or good	267 (50)	10 (18.9)	257 (53.4)	1.00	
fair or bad	267 (50)	43 (81.1)	224 (46.6)	5.18 (2.52–10.64)	<0.001
Use of emergency services in the past year					
No	469 (88.0)	45 (84.9)	424 (88.3)	1.00	
Yes	64 (12.0)	8 (15.1)	56 (11.7)	1.42 (0.64–3.13)	0.385
Hospitalized in the past year					
No	487 (91.4)	44 (83.0)	443 (92.3)	1.00	
Yes	46 (8.6)	9 (17.0)	37 (7.7)	2.35 (1.06–5.22)	0.037
Somatic climacteric symptoms					
No	323 (60.6)	25 (47.2)	298 (62.1)	1.00	
Yes	210 (39.4)	28 (52.8)	182 (37.9)	1.89 (1.07–3.34)	0.029
Psychological climacteric symptoms					
No	306 (57.4)	23 (43.4)	283 (59.0)	1.00	
Yes	227 (42.6)	30 (56.6)	197 (41.0)	1.51 (0.79–2.91)	0.217

Figures may not add up due to weighting

% are column percentages except in the header row where they are row percentages

^a^NT$, New Taiwan dollar. 10,000 NT$ approximately equal to 320 US dollars

Table 2 shows the results of the multivariate logistic regression analysis. Seven variables were included in the final model. An educational level of elementary school or below (adjusted OR = 3.19, *P* = 0.003), nulliparity (adjusted OR = 8.10, *P* = 0.001), living alone (OR = 5.47, *P* = 0.003), never having worked (adjusted OR = 4.14, *P* = 0.008), lack of regular exercise(OR = 3.01, *P* = 0.003), a perceived fair or bad health status (adjusted OR = 4.34, *P* < 0.001), and somatic climacteric symptoms (adjusted OR = 2.32, *P* = 0.012) were found to be significant independent factors associated with depressive symptoms in late middle-aged Taiwanese women.

**Table 2:** Multiple logistic regression analysis of depressive symptoms in Taiwanese women aged 50 to 65 years (N = 533).

Variable	Adjusted odds ratio (95% CI)	*P* value
Educational level		
Secondary school or above	1.00	
Elementary school or below	3.19 (1.47–6.91)	0.003
Nulliparity		
No	1.00	
Yes	8.10 (2.38–27.60)	0.001
Living arrangement		
Not living alone	1.00	
Living alone	5.47 (1.80–16.59)	0.003
Working status		
Working now	1.00	
Worked in the past	1.39 (0.68–2.81)	0.365
Never worked	4.14 (1.46–11.72)	0.008
Regular exercise		
Yes	1.00	
No	3.01 (1.45–6.27)	0.003
Perceived health status		
Extremely good, very good, or good	1.00	
fair or bad	4.34 (1.97–9.60)	<0.001
Somatic climacteric symptoms		
No	1.00	
Yes	2.32 (1.21–4.48)	0.012

Hosmer and Lemeshow test, *P* = 0.471

## Discussion

In this secondary data analysis of the NHIS, women aged 50–65 years with depressive symptoms were significantly associated with seven socio-demographic and health-related factors. We also found 9.9% of the women had experienced depressive symptoms in the past week. In comparison, a secondary data analysis study based on a Taiwan population-based survey conducted in 2002 showed a prevalence of 4.7% in depressive symptoms among women aged 40–55 years [21]. Another study of Taiwanese women aged 45–60 years recruited from a medical center and residential community found a prevalence of depressive symptoms to be 38.7% [20]. A household survey in Hong Kong found a prevalence of clinically relevant depressive symptoms, assessed by the Patient Health Questionnaire (PHQ-9), among women aged 45–64 years to be 11.0% [18]. Such a wide range of prevalence may be attributed to variations in measurement scales, cut-off values for depressive symptoms, and sources of study subjects.

Our finding on the association between somatic climacteric symptoms and depressive symptoms is in congruence with those of prior investigations. The Harvard Study of Moods and Cycles reported a two-fold increase in the risk of developing depressive symptoms during perimenopause with women who experienced vasomotor symptoms (hot flashes) [27]. The relationship between hot flashes and depression is not yet fully understood, but it has been postulated to be mediated through severe sleep disruption and its effect on quality of life [28]. A systematic review of the association between vasomotor symptoms and depressive symptoms during perimenopause concluded that the association between the two was bidirectional. The authors suggested that vasomotor symptoms and depressive symptoms could be connected via a shared biological pathway, possibly through estradiol levels and other psychological factors [29]. However, in the present study, only somatic but not psychological climacteric symptoms were associated with depressive symptoms. In other words, the presence of psychological symptoms such as anxiousness, dysphoria, panic attacks, feeling depressed, and forgetfulness alone did not appear to increase the risk of depressive symptoms. This finding is not too surprising since the CES-D 10 was used to assess overall depressive symptoms whereas the variable “psychological climacteric symptoms” focused only on climacteric-related psychological symptoms. The Spearman’s correlation coefficient between “depressive symptoms” and “psychological climacteric symptoms” was only 0.039 (*P* = 0.384), indicating the two variables were not correlated.

Consistent with previous studies, we found that a lower educational level was associated with an increased risk of depressive symptoms in women. The 2009 Korean Community Health Survey reported an adjusted odds ratio of 2.70 (95% CI = 2.49–2.93) in women who had received no education compared with those with college degrees [30]. This association can be explained by the fact that individuals with a higher educational level generally have greater feelings of personal control and better social support, which is an important means for effectively responding to stressful life events when they occur [31].

Regarding the findings on living arrangements, a cross-sectional study based on Hong Kong census data consisting of 2003 elderly Chinese people observed that living alone was an independent risk factor contributing to depression among older Chinese women [32]. Results from the univariate analysis of our study showed that being single (divorced, unmarried, or widowed) (OR = 2.12, *P* = 0.015) and living alone (OR = 2.70, *P* = 0.003) were associated with an increased risk of depressive symptoms. However, only living alone was retained in the multivariate model. Although marital status is often a good predictor of living arrangements, the two are not the same measure. In our study, 21.8% of the women were divorced, unmarried, or widowed, but only 22.4% of these women were living alone. In other words, the other 77.6% might be living with someone who could provide them with a sense of social support. Lack of social support has been suggested as one of the main reasons for feeling lonely, which is linked to mental health [33]. Additional studies are needed to investigate whether social isolation or a subjective feeling of loneliness is a true predictor of depressive symptoms in late middle-aged women.

Nulliparity was found to be associated with an 8-fold increase in the risk of depressive symptoms in this study. Previous research has suggested a positive correlation between parity and depressive symptoms. Results from the multivariate analysis of a large cohort study with 51,088 French postmenopausal women showed that a higher number of full-term pregnancies was associated with a decreased risk of severe depressive symptoms [34]. A Norwegian register-based study indicated that childlessness was associated with a higher usage of antidepressants in late mid-life. The authors suggested that having offspring might confer a sense of meaning in life, better integration into the community, and emotional support from children [35]. In fact, perceived social support is thought to be a better protector of mental health than actual social support [36]. A positive perceived social support in caring and sick care has been shown to inhibit the onset of depressive trajectories in a 12-year longitudinal study of Taiwanese women aged 50 years and over [37].

While evidence exists that levels of job strain and decision latitude can impact the development of depressive symptoms over time [38], our study showed that simply categorizing one’s paid work status (currently working, having worked in the past, and never having worked) could yield observations for different associations with depressive symptoms. Compared with women currently employed in a paid position, those who had never worked showed a four-fold increase in the risk of depressive symptoms. In the 2006 cross-sectional baseline survey of the Korean Longitudinal Study of Aging, unemployment was significantly associated with depressive symptoms, as measured with the 10-item CES-D scale, among women aged 45–64 years [39]. While it is possible that individuals with depressive symptoms could lead to unemployment, the variable “never having worked” should not be affected by reverse causality. In addition, no significant differences in depressive symptoms were observed between currently working women and women who have worked in the past. Therefore, any history of employment would appear to be protective against depressive symptoms. One possible explanation might be that employment can improve self-esteem and social support [40] and better social support can, in turn, be associated with a low risk of depression [41].

Findings from our study also indicated that lack of regular exercise was an independent factor of increased risk for depressive symptoms in late middle-aged women. This result is consistent with previous research that depressive symptoms were negatively correlated with physical activity in women [42] and that regular physical activity reduced depressive symptoms, most notably among mildly depressed women [43]. A Cochrane review, based on 35 randomized controlled trials, also concluded that exercise was moderately more effective than a control intervention for reducing symptoms of depression [44]. A cross-sectional study of 648 middle-aged Korean women indicated that the severity of depression in subjects who exercised more than three times a week was significantly lower than in those who did not exercise [17]. Therefore, together with the findings from our study, regular physical activity should be recommended to reduce the risk of depression in late middle-aged women.

The single-item perceived health status question has been demonstrated to have adequate convergent and discriminant validity, as well as adequate reliability and sensitivity [45]. In this study, we found that a fair or poor perceived health status was significantly associated with a four-fold increase in the risk of depressive symptoms. While a number of health-related variables including hypertension, diabetes, hospitalization in the past year, and perceived health status were significantly associated with depressive symptoms in the univariate analysis, the only variable retained in the multivariate model was perceived health status. The perceived health status variable appeared to have good predictive validity in relation to depressive symptoms compared with other health-related variables, suggesting its use in future surveys to assess the overall health of an individual. Longitudinal studies have also shown that self-rated health status was associated with depressive symptoms. A 7-year cohort study of Japanese aged 40–69 years showed an increased risk of depressive symptoms in women who had a poor or very poor health (adjusted OR = 2.39, 95% CI = 1.09–5.24), adjusting for age, body mass index, area of residence, education, occupation, social network, chronic disease, sleep duration, smoking, alcohol consumption, and physical activity [46]. Another 8-year population-based prospective study also found that a poor or very poor health status was one of the significant risk factors (adjusted hazard ratio = 2.60, 95% CI = 1.31–5.14) for incident depressive symptoms, assessed by the 20-item CES-D, among German elderly individuals [47].

A few methodological limitations of our study should be acknowledged. First, all measures were based on self-reporting, which is an inherent limitation of the data available. Second, the cross-sectional design of our study limits any causal inferences from our observed associations. This highlights the need for prospective studies to allow an assessment of the direction of the temporal relationship. Third, the risk of suffering from depression or depressive symptoms is known to increase during the transition to menopause [48]. However, the questionnaires of the NHIS did not include variables that allowed for the identification of this period.

## Conclusions

In conclusion, despite the aforementioned limitations, the present study suggests independent associations of somatic climacteric symptoms, and a number of socio-demographic and health-related factors with depressive symptoms in late middle-aged Taiwanese women. Given the prevalence and adverse consequences of depression in late middle-aged women, clinicians should be vigilant for its presence among women with somatic climacteric symptoms, in particular, among those who are less educated, nulliparous, live alone, have never worked, do not exercise regularly, and have a fair or bad perceived health status.

## Abbreviations

AOR: Adjusted odds ratio
CAPI: Computer-assisted personal interviewing
CES-D: Center for epidemiologic studies short depression scale
CI: Confidence interval
NHIS: National health interview survey
OR: Odds ratio
PHQ-9: Patient health questionnaire
